# Mental health response to the COVID-19 pandemic in Kenya: a review

**DOI:** 10.1186/s13033-020-00400-8

**Published:** 2020-08-18

**Authors:** Florence Jaguga, Edith Kwobah

**Affiliations:** Moi Teaching & Referral Hospital, P.O. BOX 3-30100, Eldoret, Kenya

**Keywords:** COVID-19, Mental health, Response, Kenya, Sub-saharan africa

## Abstract

**Background:**

The COVID-19 pandemic has exerted considerable impact on public mental health globally. With the pandemic rapidly rising in sub-Saharan Africa including Kenya, there is need to provide evidence to guide the mental health response in the region.

**Objectives:**

The objective of this review is (1) to describe the mental health response to the COVID-19 pandemic in Kenya, guided by the Mental Health Preparedness and Action Framework (2) to offer context specific recommendations for improvement of the mental health response in Kenya. Such information could be useful in decision-making in Kenya as well as in the greater sub-Saharan Africa region.

**Methods:**

This narrative review is based on information obtained from official government documents released from 13th March 2020, the beginning of the pandemic in Kenya, up to 31st July 2020.

**Discussion:**

The COVID-19 response in Kenya has no formal mental health response plan. There is an unmet need for psychological first aid in the community. While guidelines for the management of mental health conditions during the COVID-19 pandemic have been prepared, implementation remains a major challenge due to a poorly resourced mental health system. There is no mental health surveillance system in place limiting ability to design evidence-based interventions.

**Conclusion:**

We propose four key strategies for strengthening the mental health response in order to mitigate the harmful impact of COVID-19 on public mental health in Kenya: (1) preparation of a formal mental health response plan specific to the COVID-19 pandemic with allocation of funding for the response (2) training of community health workers and community health volunteers on psychological first aid to enable access to support for those in need during the pandemic (3) scaling up of mobile health to increase access to care (4) conducting systematic and continuous text message surveys on the mental health impact of the COVID-19 pandemic in order to inform decision-making.

## Background

The ongoing coronavirus disease of 2019 (COVID-19) outbreak which was declared a pandemic in March 2020 [1], has exerted a substantial negative impact on the health and socio-economic structures of countries across the globe [2]. The disease, caused by a novel severe acute respiratory coronavirus 2 (SARS-CoV-2) has infected close to 17 million people and resulted in over 600,000 deaths globally [3]. Disease mitigating measures such as quarantine, isolation, curfews, lockdowns and travel restrictions have resulted in loss of income, disruptions to daily routines and social isolation [4] laying the ground for negative mental health outcomes among societies.

The World Health Organization (WHO) reports that by far, the largest public mental health impact has been in the form of stress and anxiety, and predicts a rise in depression, suicide and substance use in the coming days [5]. According to a recently developed emotional epidemic curve [6] without adequate mitigation measures, countries will experience two peaks of negative mental health consequences. The first peak is dominated by anxiety and corresponds to the peak in COVID-19 cases. A second peak of negative mental health outcomes comprising of Post-Traumatic Stress Disorder (PTSD), depression, suicide, complicated grief bereavement, and relapse of those with existing disorders, corresponds to the post-pandemic period [6]. Given the substantial anticipated burden of mental disorders in the context of the COVID-19 pandemic, it is important that the mental health response is given high priority.

The recently developed Mental Health Preparedness and Action Framework (MHPAF) [6] provides a useful schema for evaluating and guiding the mental health response during the COVID-19 pandemic. The framework was recently developed by mental health professionals drawn from all six WHO regions after realization that the WHO-Global Influenza Preparedness Plan (WHO-GIPP) [7] had no mental health component. The MHPAF is comprised of five interrelated components: (1) preparation and co-ordination; (2) monitoring and assessment; (3) reducing mental health distress and misinformation; (4) sustainability of mental health care services and (5) communication [6]. According to the framework, ‘preparation and co-ordination’ involves developing a mental health response plan, creating COVID-19 specific mental health services and training of healthcare workers on psychological first aid. Psychological first aid is a supportive response that involves offering practical support to persons who are suffering or facing crises. It involves assessing and addressing basic needs such as food, water and information. It further entails listening to people, helping them calm down and protecting them from further harm [8]. ‘Monitoring and assessment’ requires the development of a mental health surveillance system to enable continuous collection of data on mental health for at risk populations as well as the general population. A second role of the mental health surveillance system is ‘reducing mental distress due to misinformation’. This includes monitoring various media platforms for myths and countering them by spreading accurate information. Finally, ‘sustainability of mental health care services’ entails the allocation of adequate funds to help mitigate the burden of mental health disorders during and after the pandemic [6].

The cases of COVID-19 are rapidly rising in the sub-Saharan Africa with concerns being raised about its capacity to deal with the pandemic [9]. Kenya, a Low Middle Income Country in Eastern Africa, was listed as being at high risk for importation of COVID-19 at the beginning of the pandemic in Africa [9, 10]. In addition, Kenya has been reported as having a low Infectious Disease Vulnerability Index (IVDI) indicating its high vulnerability to the outbreak due to fragile health systems [9]. As at 31st July 2020, Kenya has 21,354 confirmed cases of COVID-19 and 377 deaths [11]. A 21 member national emergency response committee has been set up and is responsible for overseeing and coordinating the overall COVID-19 response [12] guided by the National 2019 Novel Coronavirus Contingency (Readiness and Early Response) Plan [13]. The plan however offers no guidance on a mental health response, despite direction from the Kenya Mental Health Policy 2015–2030 [14] requiring that mental health care is provided during and after disasters.

The Ministry of Health (MOH) through its Division of Mental Health has nonetheless embarked on efforts to deliver mental health care during the pandemic. Unfortunately, the mental health response is occurring against a backdrop of an under-resourced mental health care system characterized by inaccessible services, an acute shortage of mental health workers and limited funding [15]. This, coupled with the lack of a formal mental health response plan is hindering current efforts aimed at mitigating the mental health impact of COVID-19 in the community. Given the rising incidents of domestic violence [16] and alcohol use [17] during the COVID-19 pandemic in Kenya, and the importance of behavioral strategies in containing the pandemic, a stronger mental health response is warranted.

The aim of this review is therefore (1) to provide an overview of the mental health response to COVID-19 by the Government of Kenya guided by the MHPAF. A literature search revealed no paper describing the mental health response to COVID-19 in a sub-Saharan Africa country [2] to offer context specific recommendations for improvement of the mental health response in Kenya. Such information could be useful in guiding the mental health response in Kenya and in other sub-Saharan Africa countries.

## Methods

The aim of this narrative review is to describe the mental health response to COVID-19 by the Kenyan government. Documents for review were identified following consultation with experts working at the Division of Mental Health at the MOH. We additionally hand-searched the websites of the MOH and other ministries that were listed by the government as being key in the COVID-19 response [13] for documents and web-pages with content relevant to the five components of the MHPAF. In total, 11 documents released between 13th March 2020 when the first case of COVID-19 was announced in Kenya, and 31st July 2020 were identified and included for review (Table 1).

**Table 1:** Documents included in this review

Author	Document and year
MOH	National 2019 Novel Coronavirus Contingency (Readiness and Early Response) Plan [13]
	Psychological first aid guide for COVID-19 response in Kenya, 2020 [8]
	Kenya Situation Report of 14th May, 2020 [18]
	Interim guidance on continuity of mental health services during the COVID-19 pandemic, 2020 [19]
	A comprehensive guide to mental health and psychosocial support during the COVID-19 pandemic, 2020 [20]
	Frequently asked questions about COVID-19 [21]
	Public mental health education handout [22]
	COVID-19 outbreak in Kenya daily situation report—132 [23]
	COVID-19 mental health messages for healthcare workers [24]
	Standard operating procedures for counselors and psychologists providing mental health and psychosocial support for the COVID-19 response in Kenya [25]
Ministry of Interior and Co-ordination of National Government	The National Disaster Response Plan [26]

### Preparation and co-ordination

According to the MHPAF [6], the mental health response during the early phases of the pandemic should focus on the preparation of a mental health response plan to serve as a guide for actions to be taken during the pandemic. The ‘National 2019 Novel Coronavirus Contingency (Readiness and Early Response) Plan [13] has no provision for a mental health response. The National Disaster Response Plan, the Kenyan government’s blueprint for disaster management [26] has operational objectives relating to mental health. However, some objectives are not specific to the COVID-19 context (for example the plan emphasizes the need to ensure access to social activities such as religious activities and schooling), limiting its applicability during the current pandemic.

In order to manage the distress and anxiety often witnessed during pandemics, the MHPAF [6] recommends that health care workers are trained on how to administer psychological first aid during the early phases of the pandemic. At the beginning of the COVID-19 pandemic in Kenya, the Division of Mental Health at the MOH prepared a guide for psychological first aid [8]. Training of healthcare workers on psychological first aid using the guide is ongoing via virtual platforms. A health sector situational report on COVID-19 as at 14th May however indicated that there was an unmet need for psychological first aid in the community [18].

Lastly, ‘preparation and co-ordination’ entails the setting up of COVID-19 specific mental health services. In Kenya, a number of guidelines have been developed by the Division of Mental Health in partnership with professional bodies such as the Kenya Psychiatric Association to ensure provision of COVID-19 specific mental health services. The ‘Interim guidelines for managing mental health conditions during the COVID-19 pandemic’ [19] is one such document. It provides instruction on the in-patient and out-patient management of persons with mental health and substance use disorders who test positive for COVID-19. The document additionally outlines the delivery of mental health care for persons in isolation and quarantine, and offers guidance on how to conduct telepsychiatry. In order to ensure continuity and accessibility of mental health services, the same guidelines provide direction on the management of persons with newly diagnosed mental illness and the continued care for those with pre-existing mental illness during the pandemic. The guidelines recommend that health care facilities constitute mental health response teams in order to co-ordinate care during the pandemic [19].

Two other documents that have been prepared by the Division of Mental Health include a guide for health care workers on how to offer mental health and psychosocial support to the public [20]; and standard operating procedures for psychologists and counselors to ensure standardized delivery of psychosocial interventions during the pandemic [25].

A major challenge likely to be faced despite the guidelines is that the number of mental health facilities and mental health workers available in Kenya is scarce. For example the psychologist to population ratio is 1:4,600,000. In addition, less than 1% of the public sector health care facilities offer any form of mental health care [27]. Using mobile phones to deliver mental health services in Kenya during the current pandemic, could potentially overcome the challenges of limited infrastructure as well as ensure compliance with pandemic containment measures. Such a strategy is likely to be feasible given that the country has a 91% penetration of mobile subscriptions. In addition, Kenya has the highest share of internet usage from mobile phones as compared to desktops globally [28]. Currently, counseling is being conducted via mobile phone voice calls for persons in isolation and quarantine. In addition, a call centre whose aim is to offer both knowledge and psychosocial support to frontline health workers has been established [29]. There is however a lack of mobile mental health interventions targeting the general public.

### Monitoring and assessment

The MHPAF recommends the setting up of a mental health surveillance system during the early phases of the pandemic. This is to ensure systematic and continuous collection of data on mental health in order to assist with planning and designing of appropriate interventions [6]. Data collection and monitoring for the COVID-19 situation in Kenya is being conducted by the government and daily situational reports are provided by the MOH. None of the parameters reported relates to mental health [23], a manifestation of a Health Information System in Kenya that does not prioritize collection of data on mental health [30]. Without a system for continuous collection of mental health data, a clear understanding of the burden and risk factors of mental health problems during the current pandemic remains elusive. This may result in a poorly co-ordinated response with inefficient use of the scarce resources.

Currently in Kenya, research studies seem to be the main strategy for collecting and analyzing information on mental health during the COVID-19 pandemic. The authors are aware of on-going online studies that are being conducted by mental health professionals to investigate the burden of mental health conditions among health care workers.

### Communication and reducing the mental distress due to misinformation and ‘myths’

An additional role of the mental health surveillance system is the dissemination of accurate and timely information and to address myths surrounding COVID-19. This has been shown to reduce anxiety and stress during pandemics [6]. According to the MHPAF some of the information that ought to be relayed to the public includes strategies for promoting and preventing mental health problems, available mental health services and regular updates on the state of the pandemic [6].

In Kenya, the Ministry of Information Communication & Technology (ICT) has been tasked with communication functions during the pandemic with focus on dissemination of information on COVID-19, public education and dissemination of health messages [13]. To achieve this, the ministry has set up toll free lines and hotlines through which the public may call to receive information [31]. In addition, the Ministry of ICT in conjunction with the MOH has set up a call center whose aim is to provide information on current practices on COVID-19 to health care workers [29].

The MOH has been involved in providing information on COVID-19 to the public. Specifically, the MOH provides daily situation reports on COVID-19 which usually detail the number of cases, deaths, recoveries and offers key public health messages [23]. The MOH has in addition prepared handouts on frequently asked questions about COVID-19 [26], public mental health education [27] and COVID-19 mental health messages for healthcare workers, for online dissemination [24]. Further, the MOH has partnered with a local mobile service provider to provide accurate information on COVID-19 to the general public via a 24-hour call center. The call center has incorporated the services of medical doctors who offer assistance with technical questions [32, 33].

### Sustainability of Mental Health Care Services

Direct funding to the mental health response could not be ascertained. Given the lack of a formal mental health response plan, it is likely that funding for the mental health response is erratic and inadequate.

### Post-pandemic period

In the post-pandemic period, Kenya should conduct a thorough evaluation of the five components of a mental health response as outlined in the MHPAF. Lessons learnt should be utilized in preparing a well co-ordinated mental health response plan specific for pandemic situations.

## Conclusions and recommendations

Kenya has made attempts at instituting a mental health response to the COVID-19 pandemic despite underlying systemic challenges. However, major gaps remain. The country has no formal mental health response plan, there is an unmet need for psychological first aid, access to mental health care and psychosocial support during the pandemic remains a challenge and there is no systematic collection of data on the mental health impact of COVID-19. We therefore propose four key strategies for strengthening the mental health response in Kenya:

1. Preparation of a formal mental health response plan for COVID-19 by the Division of Mental Health with allocation of funding to the response. This ought to be prepared by a team of mental health experts and guided by the five components of the MHPAF.
2. Training of community health workers and community health volunteers on psychological first aid to enable access to psychological support for those in distress during the pandemic particularly at the grassroots level. Such trainings could be administered remotely via video conferencing given the good internet coverage and smart phone penetration rates in Kenya.
3. Scaling up of mobile health to increase access to mental health and psychosocial support for the general public during the pandemic. We propose that the medical doctors manning the 24-hour call centre receive online trainings on mental health and psychological support following which the service is expanded to incorporate delivery of brief psychological interventions to the general public.
4. In order to ensure proper surveillance of the mental health situation during the current pandemic, we propose that systematic and regular surveys are conducted to allow for monitoring of the mental health impact of COVID-19 in Kenya. Major mobile service providers in Kenya have survey platforms that use text messaging. These constitute potential means through which mental health surveillance could be conducted. Given, the existing partnerships between the MOH and local mobile service providers, such an initiative is likely achievable.

## Data Availability

Not applicable.

## Abbreviations

COVID-19: Coronavirus disease of 2019
ICT: Information Communication & Technology
IVDI: Infectious Disease Vulnerability Index
MHPAF: Mental Health Preparedness & Action Framework
MOH: Ministry of Health
PTSD: Post-Traumatic stress Disorder
SARS-CoV-2: Severe Acute Respiratory Coronavirus 2
WHO-GIPP: World Health Organization Global Influenza Preparedness Plan
WHO: World Health Organisation

## References

[1] World Health Organisation Timeline—COVID-19. 2020. https://www.who.int/news-room/detail/27-04-2020-who-timeline---covid-19. Accessed 31 May 2020

[2] Nicola M, Alsafi Z, Sohrabi C (2020). The socio-economic implications of the coronavirus pandemic (COVID-19): a review. Int J Surg.

[3] World Health Organisation. Coronavirus disease (COVID-19) situation report—196. 2020. https://www.who.int/docs/default-source/coronaviruse/situation-reports/20200803-covid-19-sitrep-196-cleared.pdf?sfvrsn=8a8a3ca4_4. Accessed 4 Aug 2020

[4] Alradhawi M, Shubber N, Sheppard J, Ali Y (2020). Effects of the COVID-19 pandemic on mental well-being amongst individuals in society—a letter to the editor on “The socio-economic implications of the coronavirus and COVID-19 pandemic: a review”. Int J Surg.

[5] WHO/Europe. Coronavirus disease (COVID-19) outbreak—mental health and COVID-19. 2020. https://www.euro.who.int/en/health-topics/health-emergencies/coronavirus-covid-19/technical-guidance/mental-health-and-covid-19. Accessed 31 Jul 2020.

[6] Ransing R, Adiukwu F, Pereira-Sanchez V (2020). Mental health interventions during the COVID-19 pandemic: a conceptual framework by early career psychiatrists. Asian J Psychiatr.

[7] World Health Organization. Pandemic influenza preparedness in WHO Member States: Report of a Member States Survey. 2019. https://www.who.int/influenza/preparedness/pandemic/member_state_survey/en/. Accessed 31 May 2020.

[8] Republic of Kenya. Ministry of Health. Psychological First Aid (PSYCHOLOGICAL FIRST AID) guide for COVID-19 response in Kenya. 2020. https://www.health.go.ke/wp-content/uploads/2020/05/Mental-Health-GUIDE-FOR-COVID-19-RESPONSE-IN-KENYA_compressed-2.pdf. Accessed 31 May 2020

[9] Gilbert M, Pullano G, Pinotti F, Valdano E, Poletto C, Boëlle PY (2020). Preparedness and vulnerability of African countries against importations of COVID-19: a modelling study. Lancet.

[10] World Health Organisation. WHO ramps up preparedness for novel coronavirus in the African region. 2020. https://www.afro.who.int/news/who-ramps-preparedness-novel-coronavirus-african-region. Accessed 31 May 2020

[11] United Nations Office for the Co-ordination of Humanitarian Affairs, World Health Organisation Kenya COVID-19 operations dashboard. 2020. https://app.powerbi.com/view?r=eyJrIjoiM2NiMTk0NmItM2I3YS00MTY0LWE0NzEtOTY0OTdkMWMxNTRiIiwidCI6IjBmOWUzNWRiLTU0NGYtNGY2MC1iZGNjLTVlYTQxNmU2ZGM3MCIsImMiOjh9. Accessed 3 Aug 2020

[12] Republic of Kenya. Executive order 2 of 2020. 2020. https://www.mfa.go.ke/wp-content/uploads/2020/03/executive-order-2–2.pdf. Accessed 31 May 2020

[13] Republic of Kenya. National 2019 novel coronavirus contingency (readiness and early response) plan. 2020.

[14] Ministry of Health. Kenya mental health policy 2015–2030. 2015. https://publications.universalhealth2030.org/uploads/Kenya-Mental-Health-Policy.pdf. Accessed 30 Apr 2020

[15] Bitta MA, Kariuki SM, Chengo E, Newton CRJC (2017). An overview of mental health care system in Kilifi, Kenya: results from an initial assessment using the World Health Organization’s Assessment Instrument for Mental Health Systems. Int J Ment Health Syst.

[16] Domestic violence on the rise amid pandemic: the Standard. 2020. https://www.standardmedia.co.ke/article/2001367916/domestic-violence-on-the-rise-amid-pandemic. Accessed 31 May 2020

[17] Nacada raises alarm over rise in online liquor sales: daily nation. 2020. https://www.nation.co.ke/dailynation/news/nacada-raises-alarm-over-rise-in-online-liquor-sales-288746. Accessed 31 May 2020

[18] United Nations Office for the Coordination of Humanitarian Affairs Kenya Situation Report. 2020. Kenya Situation Report of 14th May, 2020. 2020. https://reliefweb.int/sites/reliefweb.int/files/resources/Situation%2520Report%2520-%2520Kenya%2520-%252014%2520May%25202020.pdf. Accessed 31 May 2020.

[19] Republic of Kenya. Ministry of Health. Interim guidance on continuity of Mental Health Services. 2020. https://www.health.go.ke/wp-content/uploads/2020/05/INTERIM-GUIDANCE-ON-CONTINUITY-OF-MENTAL-HEALTH-SERVICES-DURING-THE-COVID-19-PANDEMIC_compressed-2.pdf. Accessed 30 May 2020

[20] Republic of Kenya. Ministry of Health. A comprehensive guide on Mental Health and Psychosocial Support during the COVID-19 pandemic. 2020. https://www.health.go.ke/wp-content/uploads/2020/04/Final-Comprehensive-Guide-on-Mental-Health-and-Psychosocial-Support-For-COVID-19-Pandemic-4.pdf. Accessed 30 May 2020.

[21] Republic of Kenya. Ministry of Health. Frequently asked questions about coronavirus (COVID-19). 2020. https://www.health.go.ke/wp-content/uploads/2020/03/FAQs-FOR-PRESS-KIT1.pdf. Accessed 30 May 2020

[22] Republic of Kenya. Ministry of Health.Public Mental Health Education during COVID-19 pandemic. 2020. https://www.health.go.ke/wp-content/uploads/2020/03/Public-Mental-Health-AW.pdf.pdf.pdf. Accessed 30 May 2020

[23] Republic of Kenya. Ministry of Health. COVID-19 outbreak in Kenya Daily Situation report—132. https://www.health.go.ke/wp-content/uploads/2020/07/Kenya-COVID-19-SITREP-132–27-Jul-2020.pdf. Accessed 31 July 2020.

[24] Republic of Kenya. Ministry of Health. Covid-19 mental health messages for healthcare workers. https://www.health.go.ke/wp-content/uploads/2020/05/mentalhealth-02-converted.pdf. Accessed 31 July 2020.

[25] Republic of Kenya. Ministry of Health. Standard Operating procedures for psychological counselors and psychologists providing MHPSS for the COVID-19 response in Kenya. 2020. https://www.health.go.ke/wp-content/uploads/2020/05/COVID19-Standard-Operating-Procedures_compressed.pdf. Accessed 31 July 2020.

[26] Republic of Kenya. Office of the President. National Disaster Response Plan. 2009. https://www.ifrc.org/docs/idrl/857EN.pdf. Accessed 31 May 2020.

[27] Republic of Kenya. Office of the Auditor General. Performance audit report on Provision of Mental Healthcare Services in Kenya. 2018.

[28] Business Today. Kenya leads Africa in smartphone usage. 2020. https://businesstoday.co.ke/kenya-leads-africa-smartphone-usage/. Accessed 31 July 2020.

[29] Republic of Kenya. Ministry of Health. CS ICT launched COVID-19 call centre for health care workers. 2020. https://www.health.go.ke/cs-ict-launches-covid-19-call-centre-for-health-care-workers/. Accessed 31 July 2020.

[30] Meyer AC, Ndetei D. Providing Sustainable Mental Health Care in Kenya: a Demonstration Project. In: Forum on Neuroscience and Nervous System Disorders; Board on Health Sciences Policy; Board on Global Health; Institute of Medicine; National Academies of Sciences, Engineering, and Medicine. Providing sustainable Mental and Neurological Health Care in Ghana and Kenya: workshop summary. Washington, DC: National Academies Press (US); 2016. https://www.ncbi.nlm.nih.gov/books/NBK350312/26447267

[31] Republic of Kenya. Ministry of ICT, Innovation and Youth Affairs. COVID 19 hotline. 2020. https://ict.go.ke/covid-19/. Accessed 31 July 2020.

[32] Daily Nation. Safaricom: 20,000 call COVID-19 helpline daily. 2020. https://www.nation.co.ke/kenya/news/safaricom-20–000-call-covid-19-helpline-daily-286534. Accessed 31 July 2020.

[33] Saraficom. Doctor on call: telemedicine takes its place in a pandemic. 2020. https://newsroom.safaricom.co.ke/doctor-on-call-telemedicine-takes-its-place-in-a-pandemic/. Accessed 31 July 2020.

